# Short and Medium Chain Fatty Acids and Their Derivatives as a Natural Strategy in the Control of Necrotic Enteritis and Microbial Homeostasis in Broiler Chickens

**DOI:** 10.3389/fvets.2021.773372

**Published:** 2021-12-14

**Authors:** Luis-Miguel Gomez-Osorio, Veronica Yepes-Medina, Anne Ballou, Manuela Parini, Roselina Angel

**Affiliations:** ^1^Alura Animal Health and Nutrition, Bogotá, Colombia; ^2^Okuo, Medellín, Colombia; ^3^Iluma Innovation Labs, Durham, NC, United States; ^4^Silo International, Firenzi, Italy; ^5^Department of Animal and Avian Sciences, University of Maryland, College Park, MD, United States

**Keywords:** intestinal homeostasis, intestinal health, medium-chain fatty acids, short-chain fatty acids, alpha-monoglycerides, clostridium perfringens, necrotic enteritis, antibiotic growth promoters

## Abstract

The use of antibiotic growth promoters (AGPs) has historically been the most important prophylactic strategy for the control of Necrotic Enteritis (NE) caused by some *Clostridium perfringens* toxin types in poultry. During the last five decades, AGPs have also been supplemented in feed to improve body weight gain and feed efficiency as well as to modulate the microbiome (consisting of microbes and their genes both beneficial and potentially harmful) and reduce enteric pathogens, among other benefits. New regulatory requirements and consumer preferences have led to strong interest in natural alternatives to the AGPs for the prevention and control of illnesses caused by enteric pathogens. This interest is not just focused on the direct removal or inhibition of the causative microorganisms but also the improvement of intestinal health and homeostasis using a range of feed additives. A group of promising feed additives is short- and medium-chain fatty acids (SCFA and MCFA, respectively) and their derivatives. The use of SCFA and MCFA, including butyric, caproic, caprylic, capric, and lauric acids, has shown strong effects against NE in broilers both at experimental and commercial levels. These fatty acids also benefit intestinal health integrity and homeostasis. Other effects have also been documented, including increases in intestinal angiogenesis and gene expression of tight junctions. Chemical modifications to improve stability and point of release in the intestine have been shown to improve the efficacy of SCFA and MCFA and their derivatives. The aim of this review is to give an overview of SCFA, MCFA and their derivatives, as an alternative to replace AGPs to control the incidence and severity of NE in poultry.

## Introduction

Necrotic enteritis (NE) is a poultry illness caused by toxin-producing strains of *Clostridium perfringens* (CP) type A, type C and type G (1). NE is a pathology of global concern, with important consequences for flock productivity and economic viability (2). The total cost of NE outbreaks worldwide has been estimated to be over 2 billion dollars annually (3, 4). NE can present itself as a sudden increase in mortality or simply as a subclinical illness (2). CP is a normal inhabitant of the poultry gastrointestinal tract (GIT), although it does not always lead to NE. As an illness, NE is multifactorial requiring certain predisposing factors for its development, including some not completely known specific diet components (5), poor management leading to stress (6), and the presence of other intestinal pathogens like coccidia (*Eimeria* spp.) (7), *Salmonella* (*Salmonella* Typhimurium) (8) and Avian Pathogenic *Escherichia coli* (9). For example, components of the diet including b-glucans, mannans, cellulose, and lignin that cannot be digested by the animal can increase the viscosity of the intestinal contents, and can promote the development of NE (10). In addition, high concentrations of diet protein of animal-origin, such as meat and (or) fish meal are associated with a higher risk of NE. The association of these diet ingredients with NE is primarily due to the higher potential for undigested nutrients reaching the lower GIT and becoming a substrate for pathogenic bacteria such as CP and altering the microbiome of the chicken (11, 12). Other dietary changes, or increases in stocking density and rapid growth can also cause stress, leading to increased inflammation and immune activity, which can make animals more susceptible to infections like coccidiosis (6, 13, 14).

*Eimeria acervulina* and *E maxima* infection make vulnerable to NE through the induction of local T cell-mediated inflammatory outcomes that enhance intestinal mucogenesis resulting in increased water, mucus content in fecal material leading to diarrhea (15). A possible relation between coccidial-induced mucogenesis and the NE outbreaks was supported in a pig model of total parenteral nutrition (16). This nutritional model induces small intestinal inflammation with concomitant increase of acidomucin goblet cells and density of mucolytic bacteria such as CP similar to intestinal coccidiosis in the chicken. Mucin, the primary protein component of mucus, is an ideal substrate for the proliferation of CP, as this bacterium has chitinase which is involved in mucine degradation (17, 18). On the contrary, mucus may exert a protection, during *in vivo* conditions, in the ceca against *Campylobacter jejuni* avoiding the bactericidal effects of medium chain fatty acids (MCFA) in Broilers (19).

Since the 1980s antibiotic growth promoters (AGPs) have been utilized widely in poultry diets to improve performance and feed conversion (20). They have also been utilized to protect animals from the adverse effects of enteric microorganisms (pathogenic and/or opportunistic) (21) as well as to modulate inflammation (22). Reports shows that 24.6 million pounds of antimicrobials are used for non-therapeutic purposes in chickens, cattle, and swine, compared with just 3.0 million pounds used for human medicine (23). Estimations by the pharmaceutical industry-sponsored Animal Health Institute are more conservative, suggesting that of 17.8 million pounds of antimicrobials used for animals, only 3.1 million pounds are used non-therapeutically (23). Antibiotics have come under increasing scrutiny by some scientists, consumers, and government regulators due to the potential development of human pathogenic multi-drug-resistant bacteria after prolonged use (24).

The control of NE in particular has been based on the use of AGPs and ionophore anticoccidials. In some regions including the United States and Asia, ionophore anticoccidials and AGPs are used in combination. Therefore, there is an urgent need to develop alternative strategies and interventions that allow for the management of NE from a control and prevention perspective.

## Etiological Agent

The etiological agent of NE is NetB toxin-producing CP, a Gram-positive non-motile rod bacillus that forms subterminal spores. It is a strict anaerobe, although, these bacteria can survive in the presence of oxygen and/or in low superoxide concentration, which makes it an anaerobic aero-tolerant bacillus (25). CP is mainly found in the environment and in the GIT of humans, mammals and birds as a part of normal intestinal microbiota (26). However, under certain predisposing conditions, CP can act as a potent pathogen causing a variety of histotoxic and enteric diseases in humans, pigs, sheep, cows, and birds (27). CP produces at least 20 different toxins (28, 29) and a new classification has been proposed into the 7 toxigenic types A to G based on the combination of 6 exotoxins known as alpha, beta, epsilon, iota, CPE (*Clostridium perfringens* enterotoxin) and NetB (toxin related to necrotic enteritis) produced by the bacteria (30). In addition to the above six toxins, CP produces several hydrolytic enzymes and other toxins including lecithinase, hyaluronidase, collagenase, dinases, sialidases (affecting sialic acid in the host cell membrane), amylase, and hemolysin (perfringolisin or PFO or theta toxin) (31).

## Pathogenicity

NE occurs when CP proliferates in high numbers in the GIT and produces extracellular toxins, resulting in necrotic lesions which causes increased mortality, rapid loss in performance as well as severe necrosis of the intestinal mucosa (32, 33). In recent years it has been suggested that the pore-forming NetB-positive CP is probably the main cause of NE in poultry (34). In fact, CP NetB-toxin has been reported to induce NE without the presence of alpha-toxin (35). The importance of NetB in NE was demonstrated when a NetB-targeted mutant of a virulent CP chicken isolate was constructed by homologous recombination and did not cause NE in an experimental chicken model and virulence was restored when the gene was reintroduced into the same strain (36). The cause of tissue damage and cell death appears to be similar to that caused by many other pore-forming toxins in that the pores formed by NetB allow the free flow of ions such as Na^+^, Cl^−^, and Ca^2+^ which can lead to osmotic cell lysis (37). However, the mechanism of cell death has not been conclusively elucidated for this toxin.

## Control of NE in Birds

Control strategies that can be utilized to manage NE include reduction of infection pressure of pathogens, boosting of the immune response and nutritional strategies using specific feed additives. Pathogen reduction strategies generally involve establishing effective biosecurity at the farm site, use of AGPs, and longer down-time between flocks as well as strict cleaning and disinfection protocols.

AGPs have been used as an effective tool to improve animal performance because they positively modify the GIT microbiota, decreasing bacterial fermentation, reducing the thickness of the GIT wall and suppressing bacterial catabolism (38). All of these mechanisms potentially lead to improved nutrient availability as well as contribute to the health and performance of the bird (20). Thus, dietary AGPs not only improve poultry growth and feed conversion ratio, can but also help control outbreaks of enteric diseases (3).

Prior to the discovery of the NetB toxin, vaccination was focused on toxins that may not have been associated with NE, e.g., α-toxin (39). Therefore, the vaccines had only limited efficacy in controlling NE. On the other hand, coccidiosis vaccination has been used to protect birds for the occurrence of NE (40, 41), given that coccidiosis is one of the predisposing factors (3). However, most NE experimental models are based on using oral inoculation of coccidia with about 70.000 and 5.000 sporulated oocyst per bird of *E. acervulina* and *E. maxima* respectively, in non-vaccinated animals at day 7 of age (42, 43). This Eimeria (*E. acervulina* and *E. maxima*) vaccination has been shown to cause mucogenesis and intestinal damage favoring the growth of CP resulting an NE lesions (15, 44). However, protection of birds against NE was partially achieved by vaccination with recombinant NetB (rNetB) or other antigen-related or combination vaccines but further research and development is needed if full protection is to be achieved (45).

## Natural Control Alternatives

Nutritional mitigation strategies have been widely used to reduce enteric diseases such as NE, with a focus on intestinal homeostasis (46). Some of the nutritional interventions that have shown potential for improving intestinal health include the inclusion in diets of short chain fatty acids (SCFA) and MCFAand their derivatives, prebiotics, probiotics, essential oils, vaccination, enzymes, and phytobiotics in poultry diets (1, 6, 47, 48).

## Short and Medium-Chain Fatty Acids

Organic and inorganic acids are widely used in both raw materials and finished animal feeds to inhibit bacterial growth as well as enteric pathogens. The mode of action of these acids is based on the basic principle that undissociated organic acids (non-ionized, more lipophilic) can penetrate the bacterial cell wall triggering an ionization of fatty acids, which results in disruption of the normal physiology of certain types of bacteria (49–51) including an increase in fluidity, solubility, permeability, and instability of the bacterial membrane (52, 53). Specifically, an *in-vitro* study showed that *E.coli* was negatively affected by the presence of acetate. In a modeling study based on the impact of weak acids (Benzoic, Nitric and Sorbic) on yeast the mechanisms of inhibition of the yeast are defined rapid diffusion of undissociated molecules through the membrane followed by these molecules once inside the cells releasing protons resulting in cytoplasmic acidification and reduced growth. In an *in vitro* study, 100% of *Pseudomonas aeruginosa* in a biofilm was killed by acetic acid (51). During these activities, the detergent properties of the fatty acids play a key role in preventing biofilm assembly (54). After that, the bacteria inner fluid leak into the outside of the cell, inhibiting growth and causing bacterial death (55).It has been proposed that the undissociated fatty acids, once they are inside of the bacteria, an intracellular acidification occurs, which adversely affects their amino acids and enzymes (56, 57). In addition, MCFA inhibit bacterial toxin production and expression of other virulence factors by interfering with signal transduction (58, 59). The effect of these acids is both bactericidal (killing) and bacteriostatic (growth-inhibiting) depending on the concentration, synergism among them, and target bacterium (60, 61). The acids most commonly used in diet supplementation for the control of microorganisms are formic acid, benzoic acid, citric acid, carboxylic acids (all SCFA) and their salts and as well as some MCFA including Caproic (C6:0), Caprylic (C8:0), Capric (C10:0) and Lauric acid (C12:0) (62, 63).

Although important health benefits of SCFA and MCFA have been identified in *in-vitro* models, direct addition of these compounds in animal feed had been limited due to their pungent odor and unpleasant taste. New products have been developed through the formation of calcium and/or sodium salt with the fatty acids or esterification of these acids prior to addition to feed. Esterification has an important advantage as the esterified SCFA and MCFA escape gastric digestion thus reaching the small intestine where they can exert their effect (61, 64). When these acids, in salt or esterified form are fed to animals, positive effects on growth performance, intestinal microbial growth and health status of the animals are seen (65). The potential effect of SCFA and MCFA without any protection would be limited because of prompt absorption and metabolism or both in the gastric area of the intestinal tract.

An important factor for an organic acid to control pathogens in the animal is the pH of the digesta in different regions of the GIT. For example, in the poultry GIT, the passage of organic acids through the proventriculus is critical because of the effect of pH on the dissociation of these acids (66). The pKa value of the acid is the pH at which 50% of the acid appears in its undissociated form (water-soluble molecule) and 50% in its dissociated form (fat-soluble molecule). This balance changes depending on the pH of the medium. The pKa value determines the capacity of an acid to get close to bacteria, as well as determining whether it is a pH-reducing or antimicrobial acid (Table 1).

**Table 1:** pKa values for different organic acids with potential antimicrobial capacity (67).

**Acid**	**Classification^a^**	**Chemical name**	**pKa**
Acetic	SCFA	Acetic acid	4.76
Propionic	SCFA	2-propanoic acid	4.88
Butyric	SCFA	Butanoic acid	4.82
Caproic	SCFA	1-Hexanoic acid	4.88
Caprylic	MCFA	1-Octanoic acid	4.89
Capric	MCFA	Decanoic acid	4.90
Lauric	MCFA	Dodecanoic acid	5.30

a *Classified as SCFA ≤ 6 carbons; MCFA ≥ 7–12 carbons*.

MCFA have a higher pKa value than other organic acids, so they are more effective in controlling microbial growth than in reducing pH. The higher the pKa value and the smaller the difference between the acid's pKa and the pH in specific area of the GIT (proventriculus, gizzard, small intestine, ceca, colon), the more the balance will shift to the undissociated form and the greater the antimicrobial effect (68). SCFA and MCFA will remain mostly undissociated in the acidic environment of the upper gastrointestinal tract (69). Undissociated free fatty acids can readily cross the cell membrane of the pathogens and exert their bactericidal activity more easily in the whole GIT and not only in the proximal area (70). Additionally, the efficacy of SCFA and MCFA may be enhanced when supplied in their monoglyceride forms. The reason for this is that these fatty acids are not absorbed in the upper intestine and would not be released as free fatty acids without lipase activity that occurs in the duodenum, and, hence, might have higher bactericidal activity in the distal part of the intestine (i.e. hindgut). Given their molecular structure and supposed undissociated character, monoglycerides of MCFA are assumed to have antibacterial potency which is less pH dependent when compared to their corresponding free fatty acids (71).

However, some studies have shown no consistent effects of SCFA and MCFA. For example, the supplementation of 400 mg per ton of feed of Lauric acid did not reduce NE occurrence when in a model of co-infection with *netB*-positive *CP* and multi-species *Eimeria* was applied to induce NE (72).

## Antimicrobial Activity of SCFA or MCFA and Their Derivatives (ALFA Monoglycerides)

Non-dissociated and non-polar acids pass more efficiently through the liposoluble membrane of the bacteria. Once inside the bacteria, the acid dissociates, releasing hydrogen ions (protons), drastically reducing the intracellular pH of the microbe. This decrease in pH causes the bacteria to try and protect itself by expelling those protons which is an energy demanding process (73). If the required expenditure of energy is high, it can lead the death of the bacteria. Concurrently, the newly dissociated acid inside the microbial cell also have antimicrobial activity by interfering with gene transcription and subsequent protein synthesis, which affects the capacity of the bacteria to multiply and its ability to infect the intestinal mucosa (24, 74).

In addition to the need to be present in their undissociated form, another characteristic of fatty acids, both in free and salt forms, is their rapid intestinal absorption, which is closely related to their solubility in the watery intestinal content. MCFA and SCFA are quite soluble and as a result tend to diffuse directly into enterocytes, showing little dependence on bile salts or other emulsifying substances (3). This rapid absorption drastically reduces the presence of acids in the intestine, especially in the lower GIT. To increase the presence of these acids in the distal portion of the GIT, high inclusion levels in the diet are required with more than 0.5–1% diet inclusion often necessary to ensure antimicrobial activity in the lower tract (75). To minimize rapid intestinal absorption in the upper intestine, encapsulation in a hydrogenated lipid matrix (3, 76) slows the release of these acids in the GIT. The action of endogenous pancreatic lipases on the lipid-based encapsulation allows for a slow release of the acids contained inside. However, it is difficult to optimize the balance between degradation of the lipid matrix along the small intestine as it will depend, in large part, on levels of lipase secreted by the animal (77, 78). The goal is to balance the rate of degradation of the encapsulation matrix such that an effective amount of acid reaches the distal intestine while minimizing the amount of acid remaining to be eliminated in the excreta (79).

**Figure 1:**
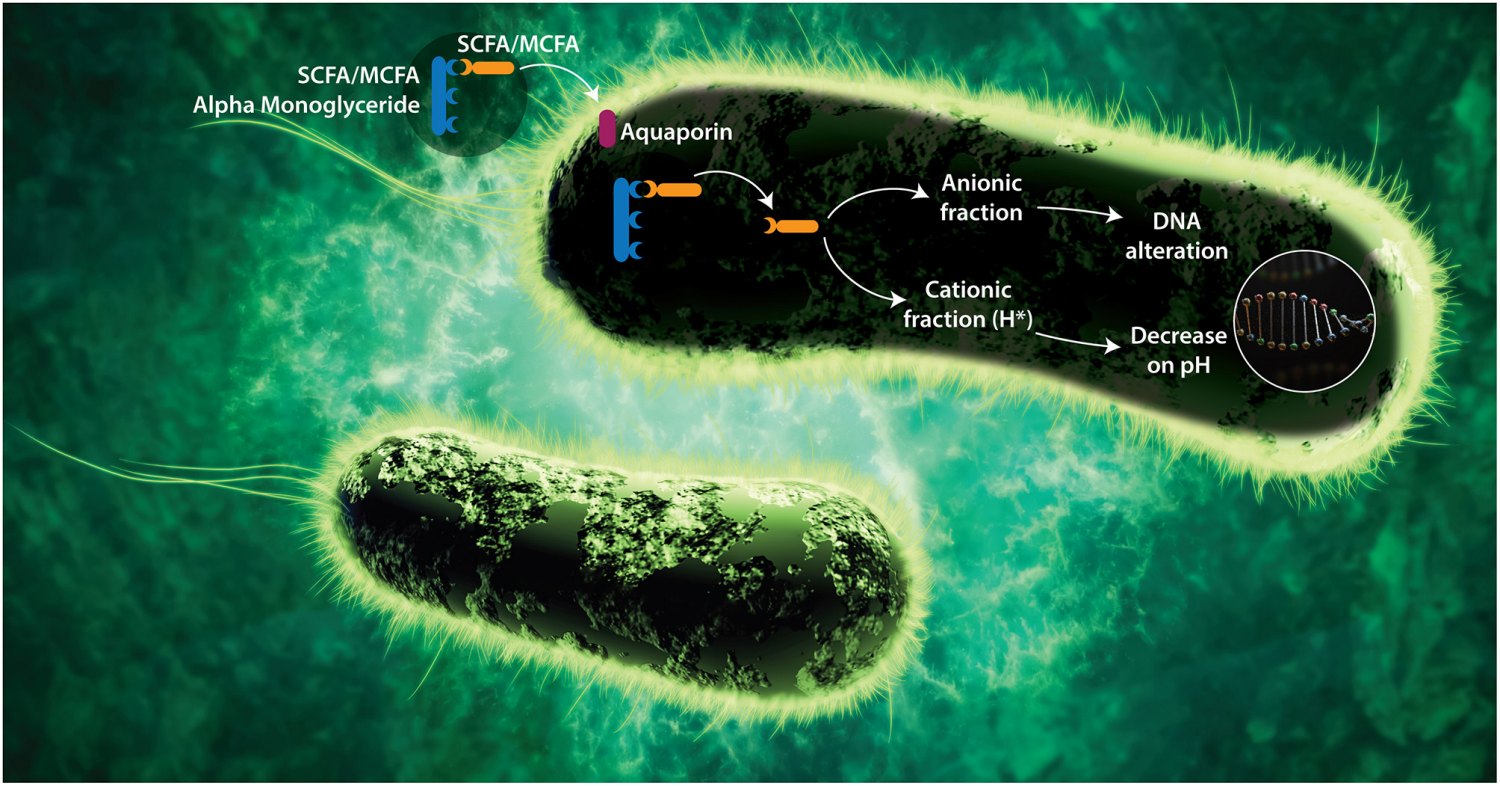
Proposed mode of action of SCFA and MCFA Monoglycerides. Alpha-monoglycerides can pass through the GIT without dissociating (as a by-pass effect) since they are linked to a glycerol. To carry out their antimicrobial functions, FA must penetrate the bacterial membrane and dissociate within the bacteria. Alpha-monoglycerides are absorbed by the bacterium through membrane proteins called aquaporins, which are selective toward glycerol due to its energetic capacity. Once they are inside the bacterium, the FA dissociates from the glycerol likely under the effect of specific enzymes (bacterial lipase), releasing its cationic (H+) and its anionic (A–) fraction (83). As the bacterium does not have a nuclear membrane, the nucleic acids are free in the cytoplasm and the anionic portion of the acid interacts directly with them, affecting the processes of translation, transduction, and replication. Bacterial pathogenesis depends on the expression of virulence factors that are encoded at the DNA, and those depend on transduction and translation mechanisms, therefore there would be a negative impact on the pathogenic capacity and on the expression of resistance mechanism in microorganisms (58, 59, 76). On the other hand, the cationic fraction (H+) of the acid decreases the internal pH of the bacterium, denaturing proteins and affecting the enzymatic activity. The catabolic activity of the enzymes depends on the pH, therefore, if the pH decreases, the isoelectric point needed for the catabolic activation is modified and the metabolism of the bacteria decreases, leading to bacteriostasis (73). To counteract the decrease in pH, the bacterium tries to remove the hydrogen ions through membrane protein complexes (ATPases) that lead to significant energy expenditure.

1-α monoglycerides (esterified adducts of a fatty acid and a glycerol molecule) can be formed with several organic acid including SCFA and MCFA. The linking of the acid to the glycerol occurs at the first position of a glycerol molecule via an ester bond. The success of alpha-monoglycerides lies in this unique molecular structure. The molecules are pH independent and less susceptible to enzymatic breakdown, which makes them active in the entire GIT. Research shows that alpha-monoglycerides have a much stronger antimicrobial effect compared to conventional organic acids (73, 80, 81). The amphipathic monoglycerides form micelles that penetrate the cell membrane and alter membrane permeability (82). This is explained by their mixed character (water and lipid soluble) leading to their entry through the bacterial membrane. SCFA 1-α monoglycerides can penetrate through the aquaglyceroporins in the bacterial wall (Figure 1). These are protein structures that act as channels allowing the entry of glycerol, which is used by the bacteria as an energy source.

MCFA 1-α monoglycerides disrupt the phospholipid membrane of pathogens forming micelles at lower concentrations than MCFAs, which helps to explain why monoglycerides are often more biologically potent than other types of SCFA and MCFA. For some bacteria the longer-chain acids or longer-chain monoglycerides may be more effective (for example, C12 may prove to be more effective than C10 or C8), but with other types of bacteria the situation may be the opposite. (84). For example, the C12 monoglyceride (glycerol monolaurate, abbreviated as GML) has a lower critical micelle concentration (CMC, defined as the concentration of surfactant above which micelles form) value (60 μmol/L at pH 7.4) and typically greater potency than both the C12 fatty acid (lauric acid; CMC of 900 μmol/L at pH 7.4) and C10 monoglyceride (glycerol monocaprate; CMC of 600 μmol/L at pH 7.4) (85). Another important consequence of MCFAs and monoglycerides targeting pathogenic membranes is that it is more difficult for susceptible pathogens to develop resistance to these compounds. It is generally acknowledged that there is a very high barrier for pathogens to develop resistance to fatty acids and monoglycerides (80, 86). This antimicrobial activity has been confirmed for different SCFA and MCFA monoglycerides, which are effective against intestinal pathogens such as *Salmonella* Typhimurium, *E.coli, Campylobacter jejuni*, and *Clostridium* spp (62, 79, 86). An interesting effect was shown with the inclusion of 3 mg/kg of Caproic acid to the feed of Broiler Chickens which suppress the expression of *Salmonella* pathogenic genes required for invasion of host cells as well as decreasing the amount of bacteria (87). It was also reported that the application of an emulsion mixture of MCFA (Caproic, Caprylic, Capric and Lauric acids) in drinking water at 0.4% vol/vol decreased the susceptibility to colonization and prevented *Campylobacter jejuni* survival (88).

Minimum inhibitory concentrations (MICs) are defined as the lowest concentration of an antimicrobial that will inhibit the visible growth of a microorganism after overnight incubation (89) and are considered the “gold standard” for determining the susceptibility of bacteria to the antimicrobials and recently has been used to test natural compounds as replacement for antibiotics. When evaluating the antimicrobial effect of various organic acids *in vitro*, MCFA showed lower minimum inhibitory concentrations (MIC) compared to other fatty acids such as butyric acid (Table 1). In an experimental *in vitro* model of NE in broilers, lauric acid, butyric acid and essential oils (eucalyptus, clove with its active ingredient eugenol and components of essential oils such as thymol, carvacrol and cinnamaldehyde) were tested individually, it was shown that lauric acid had antimicrobial activity against CP with a MIC of 0.063 mg/mL compared with butyric acid of 6.88 mg/mL (1).

When tested *in vivo* in Broiler Chickens, killing or inhibition of CP was associated with the prevention of intestinal injuries (1). These researchers also showed that butyrate did not inhibit CP, although butyrate has been reported to be a stimulant of intestinal villus growth (90). Butyrate is a preferred energy-providing substrate over glucose and glutamine for colonic epithelial cells and may account for approximately 70% of the total energy consumption of the colonocytes (91). Other documented effects of butyrate (in a Caco-2 cell culture model) are increase cell proliferation in the small and large intestine (92, 93), and enhance piglet intestinal barrier associated with its role in facilitating tight junction assembly (90). Overall, the combination of butyric acid, MCFA, and essential oils reduces the incidence of gross lesions and promotes intestinal mucosal integrity in the control of NE.

However, research has shown better results using a mix of organic acids (consisting of Formic, Lactic, Propionic, Butyric, Acetic, Citric, Sorbic and Benzoic acids) used in comparison with MCFA blends included in feed at a 0.2%. This study pointed out that organic acids improved the ceca environment, beneficial microorganism (numbers and species variety of probiotic bacteria in the ceca) more so than MCFA and that feed to gain ratio over the whole production cycle (1 to 42 d of age) was better in Broilers fed the organic acids as compared to those fed the MCFA (94).

The antimicrobial activity of organic acids and their derivatives against several gram-negative and gram-positive bacteria including CP were reported in an *in vitro* trial (62). The MICs in mg/L (±SD) of the organic acids and their derivatives against CP strain ATCC 12,915 are shown in Table 2 for butyric acid, valeric acid, and monobutyrin and monolaurin against CP (62). The results suggested that valeric and butyric acid have similar antimicrobial activity against gram-negative and gram-positive bacteria. The mode of action of these acids is based on their ability to penetrate the bacterial cell membrane and acidify the cell cytoplasm and thus inhibiting bacterial growth. *In vitro* conditions may be affected by buffering capacity of the solution that contains the free acids (Butyric and Valeric). The solution used for MIC determination in this study (41) had a low pH and, in those conditions, the acids perform well.

**Table 2:** MIC in mg/L (+SD) of various organic acids and their derivatives against *Clostridium perfringens* strain ATCC strain 12,915 (control strain recommended by the British Society for Antimicrobial Chemotherapy-BSAC) (62).

**Compound**	MIC in mg/L (±SD) against Clostridium perfringens^*^
Butyric acid	1,200 (±400)
Valeric acid	1,300 (±700)
Sodium Formate	18,800 (±7,100)
Monopropionin	11,300 (±6,400)
Monobutyrin	2,600 (±1,300)
Monovalerin	3,100 (±1,200)
Monolaurin	300 (±400)

*MIC, Minimum inhibitory concentration*.

* *Clostridium perfringens ATCC 12,915*.

Some combinations of encapsulated organic acids blended with essential oils were assessed to mitigate NE, including Malic, Fumaric, Capric, Caprylic, Caproic and Lauric acid as well as calcium Butyrate, as sources of SCFA and MCFA in Broiler Chickens. In a study, recycling litter was used as a challenge, with this litter selected from a commercial poultry flock there was clinical NE outbreaks was diagnosed (95). These researchers reported that the supplementation with calcium Butyrate plus essential oils (Cinnamaldehyde, Carvacrol and Thymol (8:1:1 parts of each) as well as a blend of organic acids (Fumaric and Citric acid) and MCFA (Capric, Caprylic, Caproic and Lauric acid) plus calcium Butyrate ameliorated the negative effects of a NE challenge on performance and mortality (95). These researchers found a dose was important as high doses were detrimental to animal performance.

The efficacy of SCFA in combination with high doses of MCFA has been shown to be more effective in improving performance parameters in a NE challenge model in Broiler Chickens. (96). Moreover, a monoglyceride blend of MCFA (Butyric, Caprylic and Capric acid) enhanced the overall feed efficiency of birds compared with the non-supplemented group in a phase dependent effect. Higher doses (0.2%) of monoglyceride MCFA supplementation appear to be beneficial in grower phase (d 10–24) and low doses (0.075%) improved the performance in the finisher phase (d 24–35) (96). In another study, a blend of Monoglyceride of MCFA (Butyric, Caprylic, and Capric acids) and buffered Formic acid, used at 0.03 to 0.05% inclusion rate in the diet, showed the potential to improve intestinal health and reduce the mortality caused by NE induced by inoculation of a blend of field isolated Eimerias (*E.acervulina, E.maxima and E.brunetti*) followed by a CP challenge (97).

Other researchers have evaluated individual MCFA monoglycerides. Valeric acid (C5) was tested in a NE challenge trial (Coccidia vaccine follow by CP) (98). They demonstrated that low doses of Valeric acid (0.5 and 1.5%) improved performance but did not decrease NE lesions. They also showed that Valeric acid positively affected the morphology of the intestinal mucosa (98). The effects of Lauric acid, a MCFA, on induced NE was not effective in reducing the incidence and severity of NE (72). This contrasts with a previous study showing that the same inclusion of Lauric acid decreased NE incidence (from 50 to 25%) compared with an uninfected untreated group (1).

## Role of the Microbiota in Control of NE

As mentioned elsewhere in this review, other microbes can have an important role in an NE mitigation and prevention strategy. The acquisition of a diverse, stable microbiome during development is very important in resisting endemic challenges such as NE. The development of a healthy microbiome can be aided in many ways through nutrition, water quality, and other management choices. While the feed can be a source of microbes for the GIT, many bacteria found in common feed substrates are not ideally suited to long term survival in the GIT. Bacteria that aid in the development of a healthy microbiome can be supplemented with probiotic feed additives. The variety of probiotics available in the poultry industry is too large to cover in this review; however, some have shown promising benefits in the reduction of the incidence and severity of NE (99, 100). In addition to supplementation through the diet, the environment can be an important factor in microbiome-mediated resistance to NE. Studies evaluating the recycling or reuse of litter in poultry barns have demonstrated lower levels of toxin-producing CP in the ileum of broilers on used litter vs. fresh (101, 102), suggesting the contribution that previous flocks can have in helping in the development of a balanced and healthy microbiota in chicks.

The extent to which other GIT microbes affect or are affected by NE is still under investigation. It has been reported that even in the absence of clinical NE, the addition of predisposing factors of NE such as high levels of fish meal and non starch polysaccharides can reduce the levels of several butyrate and lactate producing bacteria (103). These bacteria include *Lactobacillus johnsonii*, Ruminococcaceae, and *Candidatus savagella*, all of which have previously been associated with GIT homeostasis and better animal health (104, 105). It is difficult to demonstrate a causal relationship between these microbiome changes and susceptibility to CP overgrowth and NE, but the fact that these and related species normally make up a significant portion of the ileal and/or cecal microbial populations suggests that in healthy animals, they play an important role in maintaining balance between normal GIT microbial constituents (11, 106).

The impact other microbes have on the incidence of CP can be driven by several mechanisms; competitive exclusion (the competition for a particular resource or niche in the GIT), release of bacteriocins, and possibly even the interruption of CP quorum sensing in pathogenic populations. From *in vitro* studies into the quorum sensing mechanisms used by NetB-producing CP there is a strong suggestion that this is an important step in the pathogenesis of CP in poultry (107). Our understanding of quorum sensing in complex communities such as the GIT microbiome is in its infancy. Researchers have reported several instances of interspecies cross-talk and interference in quorum sensing (108, 109).

## SCFA, MCFA and the Microbiome

Though the GIT microbiome may help regulate CP pathogenesis in several ways, secretion of metabolites such as butyrate and other SCFA are likely an important part of these mechanisms. Many common GIT bacteria ferment dietary fiber and even protein and produce SCFA (110). Firmicutes such as *Faecalibacterium, Clostridium*, and Ruminococcaceae are the principal producers of SCFA in the hindgut, and *Akkermansia* and *Bifidobacterium* also produce lactate and limited quantities of butyrate (111). Cross feeding is an important factor in the production of SCFA in the GIT, and one of many ways in which changes in bacterial composition can affect the host. Certain metabolites of bacterial fermentation are substrates for other bacteria to ferment, altering the composition of the GIT metabolome in important ways. For example, acetate and lactate produced by *Bifidobacterium* and Bacteroidetes can be consumed by butyrate-producing microbes such as *Faecalibacterium* and *Roseburia* (112).

Additionally, the GIT contains a group of g-protein coupled receptors called free fatty acid receptors, capable of reacting to SCFA. This results in a variety of different responses affecting various host systems, including the secretion of GIT hormones such as peptide YY (PYY) and glucagon-like peptide 1 (GLP1) (113). These molecules are important for the proper regulation of insulin, satiety, and appetite stimulation. Additional downstream impacts of SCFA-free fatty acid receptor (FFAR) signals include various inflammatory and immune responses (114).

SCFA, specifically butyrate, and MCFA do not typically exert strong effects on the microbial composition of the normal chicken GIT (115). However, in situations in which the GIT microbial homeostasis is challenged or susceptible to change, i.e. during periods of stress or pathogen challenge, supplementation with butyrate seems to reduce the impact of the challenge on the microbiota (116, 117). This suggests that SCFA can exert a homeostatic effect in the GIT microbial population. This is an important part of minimizing intestinal pathogens, as stress-related dysbiosis makes the normally resistant microbial community more susceptible to pathogenesis from normal constituents such as CP. Interestingly, supplementation with butyrate can decrease some butyrate-producing species in the chicken cecum. The authors speculate that this could be a feedback mechanism in response to the increased sodium butyrate coming into the GIT from the diet (117). This supports an increasingly recognized facet of the microbiome, a relentless movement toward stability and homeostasis. This complicated community is comprised of hundreds of individual members, each acting in conjunction with other species, the digesta (substrates) and the host to achieve a balance that allows for stable growth and colonization.

Work on MCFA and the microbiota lags considerably behind SCFA, for a few reasons. Though MCFA are nutritionally important and interact with the host in some of the same ways as SCFA, they are not products of microbial fermentation, but are in commercial diets that contain palm kernel, coconut or milk meals or fats. While SCFA, particularly butyrate, are major energy sources for enterocytes and microbes alike, MCFA are primarily metabolized by the host, in the liver (118).

In the last 25 years, the approach to research and ultimately solutions to production problems has had a reductionist approach focusing on understanding parts of the system rather than integrating parts with the whole. That is, looking at small parts of the problem disregarding in most cases the animal as a whole and as a superorganism. Recently, we have been seeing more and more insights into the whole animal universe with numerous components that interact and are the essence of whole animal health. We are just beginning this incredible journey into understanding, from a holistic standpoint, how the environment, feed, digesta, microbiome, and host interact at every turn. A new nascent knowledge on the interaction between the microbial community in the GIT and the cells of immune system affecting whole parts of the body is emerging based on the assumption that intestinal microbes may shape the course of inflammatory illnesses such as autoimmune human diseases like Crohn's disease and Multiple Sclerosis (119). However, outside influences or runaway growth of a few members can upset this balance, the pressure of other community members works constantly to return the community to a balance that favors as many members as possible, rather than a few opportunistic species. The question that arises is if the microbiome differs because animals that are sick or is it different because it causes disease? (120). More mechanistic studies instead of only correlational studies are needed to understand this relationship.

## Conclusions

Enteric diseases, especially NE, are a major concern in the poultry industry due to production losses, increased mortality, reduced bird welfare and increased risk of contamination of poultry products intended for human consumption. Additionally, public concern about the threat of antibiotic-resistant pathogens has forced the poultry industry to consider alternatives to antibiotic-based prevention of NE. Strategies to control NE in the absence of AGPs have focused on nutrition and biosecurity. Some promising alternatives include organic acids, SCFA, MCFA and their derivatives, probiotics, prebiotics, enzymes, plant extracts, bacteriophages, and vaccination.

The use of SCFA and MCFA in the form of alpha monoglycerides as an alternative for the control of NE has shown important results in the improvement of intestinal health and therefore in the prevention of the proliferation of pathogenic *CP* and the release of its toxins which generate strong damage in the intestinal epithelium. Although the full mechanism of action of SCFA and MCFA is not well-known, broad-spectrum activity has been demonstrated against gram positive and gram-negative bacteria such as *Salmonella, Campylobacter*, and *Clostridium* spp, making them a viable solution to reduce the use of AGPs. They also have synergistic effects when used together and can thus reduce the magnitude and duration of treatments.

## Author Contributions

All authors listed have made a substantial, direct, and intellectual contribution to the work and approved it for publication.

## Conflict of Interest

Commercial companies employed several of the authors of this paper, at the time of the writing of this paper, as follows: L-MG-O employed by Alura Animal Health and Nutrition, VY-M by Okuo, A. Ballou by Iluma Alliance, and M. Parini by Silo. Alura Animal Health and Nutrition is a company that sells therapeutic and prophylactic antibiotics and anticoccidials as well as natural health alternatives including SCFA and MCFA for animal husbandry. Okuo sells food safety products for processing plants. Iluma is involved in formulation services, sale of vitamin and mineral premixes as well as health products for animal agriculture. Silo sells vitamins, amino acids, fiber compounds, SCFA and MCFA. None of these companies exclusively sells SCFA and MCFA. The remaining authors declare that the research was conducted in the absence of any commercial or financial relationships that could be construed as a potential conflict of interest.

## Publisher's Note

All claims expressed in this article are solely those of the authors and do not necessarily represent those of their affiliated organizations, or those of the publisher, the editors and the reviewers. Any product that may be evaluated in this article, or claim that may be made by its manufacturer, is not guaranteed or endorsed by the publisher.
